# Effect of Fatty Acid Chain Modification on the Self-Assembly Behavior and Antimicrobial Activity of Antimicrobial Peptides

**DOI:** 10.3390/antibiotics15050518

**Published:** 2026-05-20

**Authors:** Hongyan Yang, Meiqian Luo, Yutao Min, Yehuan Zheng, Yanhua Xu, Bingchao Duan, Fei Pan, Kui Lu

**Affiliations:** 1 School of Food and Health Engineering, Zhengzhou University of Technology, Zhengzhou 450044, China; 2 Molecular Diagnostics Department, R&D Center, Autobio Diagnostics Co., Ltd., Zhengzhou 450016, China; 3 State Key Laboratory of Resource Insects, Institute of Apicultural Research, Chinese Academy of Agricultural Sciences, Beijing 100093, China; 4 College of Food Science and Engineering, Henan University of Technology, Zhengzhou 450001, China

**Keywords:** lipopeptides, antimicrobial peptides, self-assembly, molecular dynamics simulations, mechanism

## Abstract

**Background**: The overuse of traditional antimicrobial agents has accelerated the global spread of drug-resistant bacteria, posing a severe threat to global public health. **Methods**: In this work, a series of lipopeptides with varying fatty acid chain lengths were designed using the targeting antimicrobial peptide CL5 as the parental peptide. A variety of technical methods, including spectroscopic techniques, electron microscopy and computer simulation, were adopted to explore the self-assembly properties of the lipopeptides and their antimicrobial properties against Gram-positive and Gram-negative bacteria. **Results**: The results showed that lipopeptide self-assembly could be triggered by fatty acid chain modification with a carbon chain length exceeding 8 atoms, and hydrophobic interactions between fatty acid chains were the primary driving force for this process. The geometric mean of the minimum inhibitory concentrations of the lipopeptides exhibited an approximate “U”-shaped correlation with the length of the fatty acid chains. Among these lipopeptides, C8CL5–C12CL5 exhibited broad-spectrum and highly potent antimicrobial activity, with geometric means of 6.20, 5.16, and 8.00 μM against all tested bacteria, and selectivity index values of 12.26, 8.14, and 7.48, respectively. Furthermore, the lipopeptides exhibited high selectivity, rapid time-killing kinetics, as well as excellent thermal, pH and salt stability. Mechanistic studies revealed that the lipopeptides exerted antimicrobial effects through multiple pathways: disrupted bacterial cell membranes and caused the leakage of cellular contents, bound to bacterial genomic DNA, and promoted the production of reactive oxygen species. **Conclusions**: Collectively, lipopeptides modified with appropriate fatty acid chains exhibit broad-spectrum and highly effective antimicrobial activity, making them promising alternatives to traditional antibiotics for the treatment of bacterial infections.

## 1. Introduction

Food serves as the material foundation for human survival, and food safety is closely related to human health and life security. According to statistics from the World Health Organization (WHO), approximately 600 million people worldwide fall ill each year due to consumption of contaminated food, resulting in around 420,000 deaths [1]. Food preservatives play a crucial role in ensuring food safety, as they significantly reduce the incidence of microbial-induced food spoilage and foodborne diseases [2]. At present, only a limited number of natural preservatives are permitted for use in the food industry, mainly including nisin, ε-polylysine, and lysozyme. Moreover, these preservatives generally suffer from drawbacks such as low antimicrobial activity and narrow antimicrobial spectra, which are far from sufficient to meet the demands of the food industry [2]. In addition, the abuse of antibiotics in medicine, agriculture, animal husbandry and other fields has exacerbated the emergence of bacterial resistance. Drug-resistant bacteria enter the food chain via plants, animals, the environment and other pathways, posing a severe threat to global public health [3]. Thus, there is an urgent need to develop novel antimicrobial agents with unique mechanisms of action and low potential to induce bacterial resistance.

Antimicrobial peptides (AMPs) have attracted extensive attention as potential substitutes for traditional antibiotics due to their broad-spectrum antimicrobial activity, non-specific membrane-targeting mechanism, and low tendency to induce drug resistance [4,5]. Nevertheless, the clinical application of natural AMPs is hindered by their inherent limitations, including insufficient antimicrobial activity, high cytotoxicity toward mammalian cells, and poor stability under physiological conditions [6]. To address these issues, rational structural modifications of AMPs, including fatty acid modification, PEGylation, methylation, glycosylation, unnatural amino acid substitution, and conjugation with small-molecule drugs, have been extensively employed to enhance their biological activities [4,7,8].

Hydrophobicity is a key structural parameter of AMPs that modulates their insertion into bacterial membranes and thus the strength of their interaction with the phospholipid bilayer of bacterial membranes [9,10]. Previous studies have demonstrated that the antimicrobial activity of AMPs does not exhibit a completely consistent linear relationship with hydrophobicity [11]. A moderate increase in hydrophobicity can enhance peptide activity; however, excessive hydrophobicity disrupts the balance among the structural parameters of AMPs, leading to reduced antimicrobial activity. In addition, overly high hydrophobicity potentiates the interaction of AMPs with mammalian cells, inducing significant hemolysis and cytotoxicity [12]. Conjugation of fatty acid chains to the specific terminus of AMPs is one of the effective strategies for hydrophobicity modulation, which features facile synthesis, controllable hydrophobicity, and minimal disruption to the core structure of the parental peptide [13]. Moreover, fatty acid chain modification can enhance the hydrophobic interactions between AMP molecules, thereby inducing their self-assembly [14]; moderate self-assembly can increase the local density of antimicrobial active sites, improve the efficiency of targeted binding to bacterial membranes, and thus enhance the antimicrobial activity [15].

In our previous work, we obtained a lipopolysaccharide (LPS)-targeting AMP CL5 with potent inhibitory activity against Gram-negative bacteria [16]. Subsequently, we introduced a 12-carbon fatty acid chain at its N-terminus, yielding the lipopeptide C12-CL5, which exhibited broad-spectrum antimicrobial activity [17]. Covalent conjugation of fatty acid chains of varying lengths to the N-terminus of AMPs could regulate their hydrophobicity and amphipathicity, thereby promoting controllable self-assembly of lipopeptides, and ultimately modulating their membrane-disrupting activity against bacteria. In this work, a series of lipopeptides with fatty acid chain lengths ranging from 2 to 18 carbons was synthesized (Figure S1), aiming to investigate the effects of fatty acid chain length on the self-assembly properties and antimicrobial performance of the LPS-targeting AMP. The self-assembly properties of these lipopeptides were investigated using spectroscopy, atomic force microscopy (AFM), zeta potential measurement, molecular dynamics (MD) simulations, and independent gradient model (IGM) analysis. The relationship between fatty acid chain length and self-assembly capacity was analyzed, and the primary driving forces of lipopeptide self-assembly were elucidated. Key indicators including antimicrobial activity, time-killing kinetics, cytotoxicity, and stability were experimentally determined, and the relationship between fatty acid chain length and antimicrobial activity was analyzed. Furthermore, the antimicrobial mechanism of the lipopeptides was explored through scanning electron microscopy (SEM), transmission electron microscopy (TEM), confocal laser scanning microscopy (CLSM), circular dichroism (CD) spectroscopy, cytoplasmic membrane depolarization, outer membrane (OM) and inner membrane (IM) permeability assays, in combination with gel electrophoresis and reactive oxygen species (ROS) detection assays. This work not only yields a panel of lipopeptides with potent antimicrobial activity, but also provides critical insights for the rational design of broad-spectrum and highly effective AMPs.

## 2. Results

### 2.1. Characterization of Lipopeptides

The Electrospray ionization mass spectrometry (ESI-MS) and reversed-phase high-performance liquid chromatography (RP-HPLC) profiles of the lipopeptides C2CL5–C18CL5 are shown in Figure S2, and the results are presented in Table S1. The measured molecular weights of all lipopeptides were consistent with their theoretical values, and the purities exceeded 95%, which satisfied the requirements for subsequent experiments. Under identical RP-HPLC conditions, the retention times of C2CL5–C18CL5 on the chromatographic column increased with the elongation of the fatty acid chain, rising from 10.646 min to 35.262 min, which were higher than that of the parental peptide CL5 (8.485 min).

### 2.2. Self-Assembly Properties of Lipopeptides

**Figure 1:**
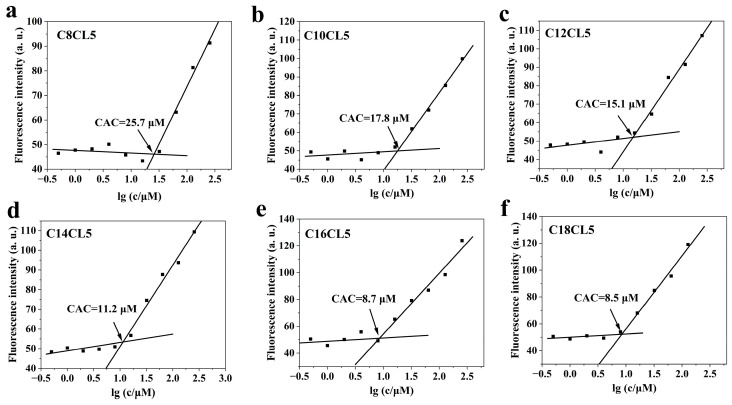
The fluorescence intensity of 1,8-ANS against the logarithmic values of lipopeptide concentrations; (**a**) C8CL5, (**b**) C10CL5, (**c**) C12CL5, (**d**) C14CL5, (**e**) C16CL5, (**f**) C18CL5. The CAC value was determined by intersection of the tangent at the inflection with the horizontal tangent through the points at low concentrations.

The hydrophobic fluorescent probe 1,8-ANS was used to evaluate the self-assembly ability of lipopeptides in the concentration range of 1–256 μM. No significant changes in the fluorescence intensity of 1,8-ANS were observed in the CL5, C2CL5–C6CL5 (Figure S3). In contrast, the fluorescence intensity of 1,8-ANS increased sharply when the concentration of C8CL5–C18CL5 reached certain thresholds. The critical aggregation concentration (CAC) values of C8CL5–C18CL5 were determined to be 25.7, 18.9, 15.1, 11.2, 8.7, and 8.5 μM, respectively (Figure 1).

C8CL5–C18CL5 all formed transparent and homogeneous systems at a concentration of 100 μM. Figure S4a shows that at 100 μM (above their respective CAC values), C8CL5–C18CL5 all exhibited a distinct negative peak at approximately 198 nm, a characteristic peak of the random coil structure. Additionally, the random coil characteristic peak was also observed for C8CL5–C18CL5 at 6 μM (below their respective CAC values), as shown in the inset of Figure S4a. Figure S4b showed that C8CL5–C18CL5 were predominantly composed of random coil structure, with relatively low proportions of β-sheet and β-turn structures, and the lowest proportion of α-helix structure.

**Figure 2:**
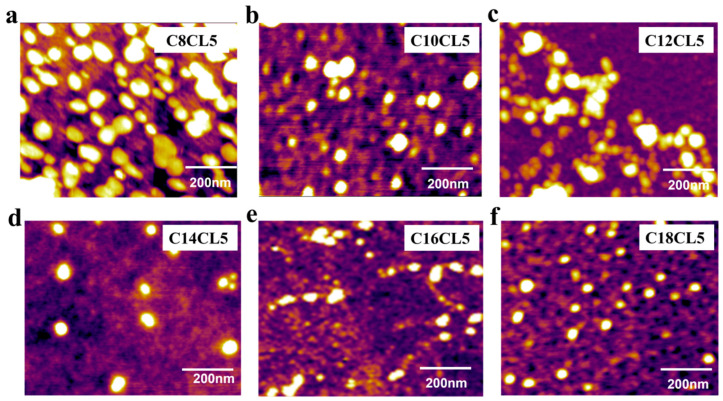
AFM images of C8CL5 (**a**), C10CL5 (**b**), C12CL5 (**c**), C14CL5 (**d**), C16CL5 (**e**), and C18CL5 (**f**).

From AFM images, C8CL5–C18CL5 at a concentration of 100 µM could self-assemble into nearly spherical nanostructures in aqueous solution (Figure 2). In contrast, C2CL5–C6CL5 with shorter fatty acid chains exhibited no obvious self-assembled morphologies (Figure S5). Figure S6 shows that the zeta potentials of C8CL5–C18CL5 were all positive, ranging from +20.72 to +43.23.

**Figure 3:**
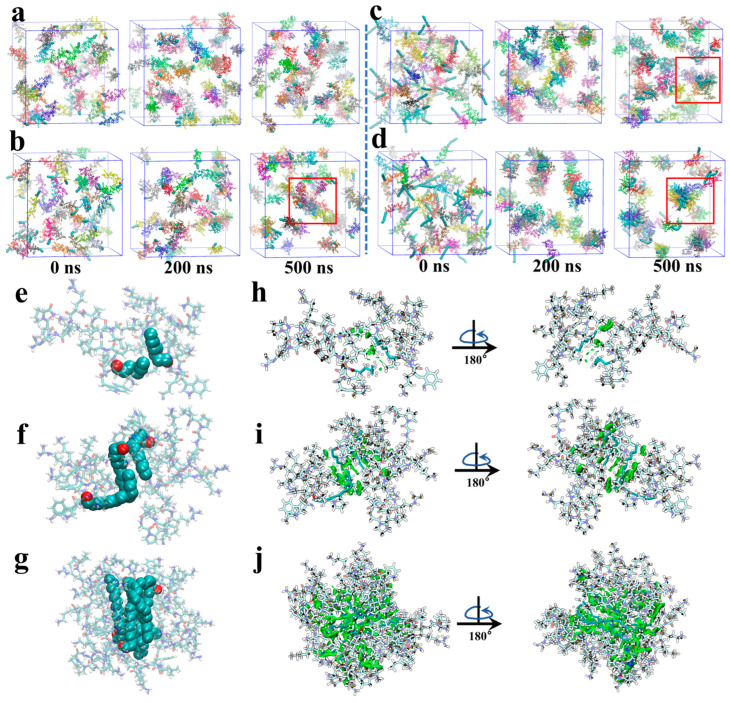
MD simulation snapshots of C4CL5 (**a**), C8CL5 (**b**), C12CL5 (**c**) and C18CL5 (**d**) at 0, 200, and 500 ns. The red square denotes the aggregate of lipopeptides. Representative structures of C8CL5 (**e**), C12CL5 (**f**), and C18CL5 (**g**) aggregates at 500 ns. The sphere models represent the fatty acid chains in the lipopeptides. Weak intermolecular interactions of C8CL5 (**h**), C12CL5 (**i**) and C18CL5 (**j**) by IGM analysis. Note: In (**a**–**g**), the hydrophobic fatty acid groups are represented by dark green sphere. Different colors of the isopotential surfaces in (**h**–**j**) represent different interactions. Specifically, green, blue, and red represent van der Waals forces, hydrogen bonds, and steric hindrance, respectively.

Short-, medium-, and long-chain fatty acid-modified peptides C4CL5, C8CL5, C12CL5 and C18CL5 were selected as representative lipopeptides, and their self-assembly behavior was investigated using all-atom MD simulations. As illustrated in Figure 3a, C4CL5 existed consistently as monomers in the simulation box throughout the 500-ns MD simulations, whereas C8CL5 exhibited observable aggregation propensity at 500-ns (Figure 3b). For the C12CL5 and C18CL5 systems, both formed relatively ordered self-assembled structures at 200 ns, and the self-assembled structures remained in a relatively stable state (500-ns) without aggregating into larger aggregates (Figure 3c,d). Figure 3e–g show representative aggregate structures of C8CL5, C12CL5, and C18CL5 at 500 ns, respectively. The fatty acid chains of these lipopeptides aggregated and remained in the interior of the assemblies, while the hydrophilic peptide chains were distributed on the outer surface. The independent gradient model (IGM) was used to analyze the intermolecular interactions in the self-assembled lipopeptides C8CL5, C12CL5 and C18CL5. As shown in Figure 3h–j, the van der Waals forces between fatty acid chains were the primary driving force for the self-assembly of these lipopeptides.

### 2.3. Antimicrobial Properties of Lipopeptides

**Figure 4:**
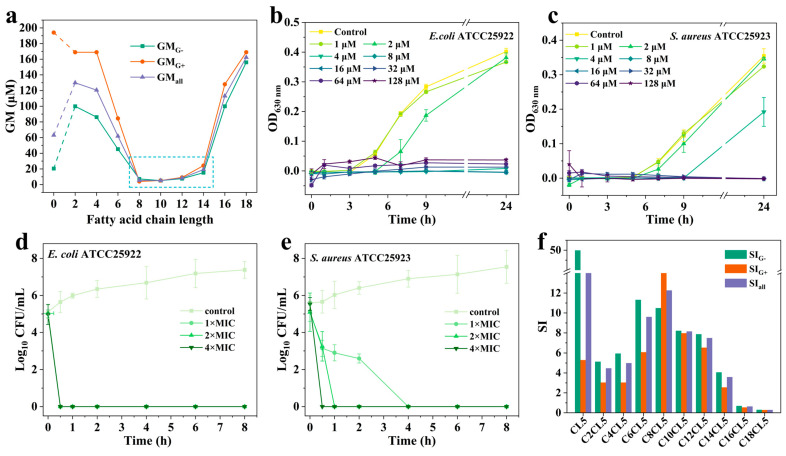
(**a**) The GM values of the MICs of the lipopeptides against Gram-negative bacteria, Gram-positive bacteria, and all tested bacteria. The blue square denotes the lipopeptides with relatively low GM values. Effect of different concentrations of C10CL5 on the growth curves of *E. coli* ATCC25922 (**b**) and *S. aureus* ATCC25923 (**c**). Time-killing kinetics of C10CL5 against *E. coli* ATCC25922 (**d**) and *S. aureus* ATCC25923 (**e**). (**f**) The SI values of the lipopeptides toward HEK-293 cells. The measurements in (**b**–**e**) were repeated three times, and the data are expressed as the mean ± SD.

As listed in Table S2, compared with the parental peptide CL5, lipopeptides C2CL5–C6CL5, C16CL5, and C18CL5 exhibited significantly reduced activity against Gram-negative bacteria, and some even lost their inhibitory activity. In sharp contrast, C8CL5–C14CL5 displayed high activity against Gram-negative bacteria, with GM_G−_ values ranging from 5.12 to 15.23 μM. C10CL5 demonstrated particularly outstanding activity (GM_G−_ = 5.12 μM), significantly outperforming CL5 (GM_G−_ = 20.49 μM). Notably, C8CL5–C14CL5 also showed good activity against Gram-positive bacteria, with GM_G+_ values ranging from 4.00 to 24.25 μM, which were significantly higher than that of PMB (64.00 μM). Overall, the GM_all_ of C8CL5–C14CL5 against all tested bacteria in this study ranged from 5.16 to 17.21 μM, and the GM_all_ value of C10CL5 (5.16 μM) was comparable to that of PMB (4.46 μM). Figure 4a demonstrates that the GM_G−_, GM_G+_, and GM_all_ values of C2CL5–C18CL5 all exhibited an approximate “U”-shaped relationship with fatty acid chain length.

The most active lipopeptide, C10CL5, was selected as the research subject to investigate its effects on bacterial growth curves. For *E. coli* ATCC25922 in the control group, the OD_630_ remained essentially unchanged within 0–3 h, increased to 0.06 at 5 h, and further rose to 0.40 at 24 h. When *E. coli* was treated with C10CL5 at concentrations equal to or greater than 1× MIC, negligible bacterial growth was observed within 24 h. In addition, when the concentration of C10CL5 was slightly below 1× MIC, *E. coli* exhibited a significantly slower growth rate than the control group, indicating that C10CL5 at the sub-MIC concentrations exerted a transient inhibitory effect and could delay its entry into the logarithmic growth phase (Figure 4b). Similar phenomena were observed in other Gram-negative bacteria (Figure S7a,b) and Gram-positive bacteria (Figure 4c and Figure S7c,d). The results of time-killing kinetic assays demonstrated that C10CL5 completely eradicated *E. coli* within 0.5 h at 1×, 2×, and 4× MIC, while the complete killing times for *S. aureus* were 4 h, 1 h, and 0.5 h, respectively (Figure 4d,e).

### 2.4. Cytotoxicity of Lipopeptides

From Figure S8, the survival rates of HEK-293 cells treated with 256 µM of the lipopeptides C2CL5–C6CL5 were 84%, 67%, and 55%, respectively. In contrast, C8CL5–C18CL5 exhibited cytotoxicity toward HEK-293 cells at higher concentrations, with IC_50_ values of 75.97, 42.01, 59.81, 61.69, 67.61, and 44.86 µM, respectively.

Based on the IC_50_ and GM values of lipopeptides, the SI values were calculated (Figure S9  and Figure 4f). For the more potent lipopeptides C8CL5–C12CL5, their SI_G−_ values against Gram-negative bacteria (SI_G−_ = 7.86–10.48) were lower than those of CL5 (SI_G−_ = 49.97), while their SI_G+_ values against Gram-positive bacteria (SI_G+_ = 6.51–18.99) were higher than those of CL5 (SI_G+_ = 5.28). The SI_all_ values of C8CL5–C12CL5 against all tested bacteria ranged from 7.48 to 12.26, indicating relatively high selectivity toward bacteria.

### 2.5. Stability of Lipopeptides

**Figure 5:**
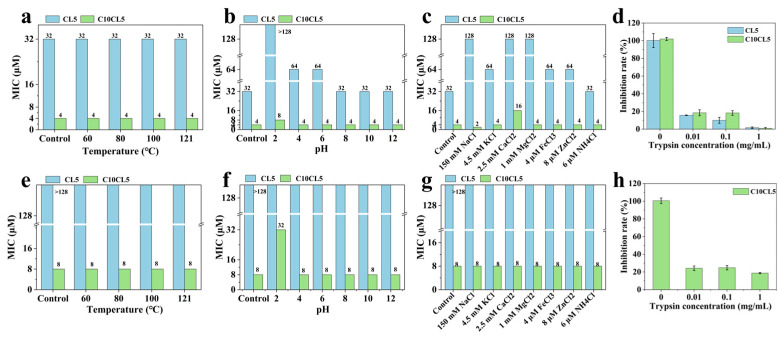
Effects of thermal, pH, physiological salts, and trypsin on the antimicrobial activity of C10CL5 against *E. coli* ATCC25922 (**a**–**d**) and *S. aureus* ATCC29523 (**e**–**h**). The untreated C10CL5 was used as the control. The measurements were repeated three times, and the data are expressed as the mean ± SD.

The stability of C10CL5, which exhibited high activity and selectivity, was investigated. After treatment at 60–121 °C for 1 h, C10CL5 exhibited activity against *E. coli* ATCC 29522 and *S. aureus* ATCC 29523 comparable to that of the untreated group (Figure 5a,e). The antimicrobial activity of C10CL5 was slightly reduced at pH 2, but remained basically unchanged under other pH conditions (pH 4–12) (Figure 5b,f). In the presence of various physiological salts, C10CL5 only showed reduced activity against E. coli in the Ca^2+^-containing system, and maintained satisfactory antimicrobial activity under other salt conditions, which was significantly better than that of CL5 (Figure 5c,g). After treatment with 0.01 mg/mL of trypsin, the activity of C10CL5 decreased markedly and further declined with increasing trypsin concentrations. When the trypsin concentration reached 1 mg/mL, C10CL5 showed negligible inhibitory effect on *E. coli*, and the inhibition rate against *S. aureus* dropped to approximately 15% (Figure 5d,h).

### 2.6. Effects of Lipopeptides on Bacterial Membranes

**Figure 6:**
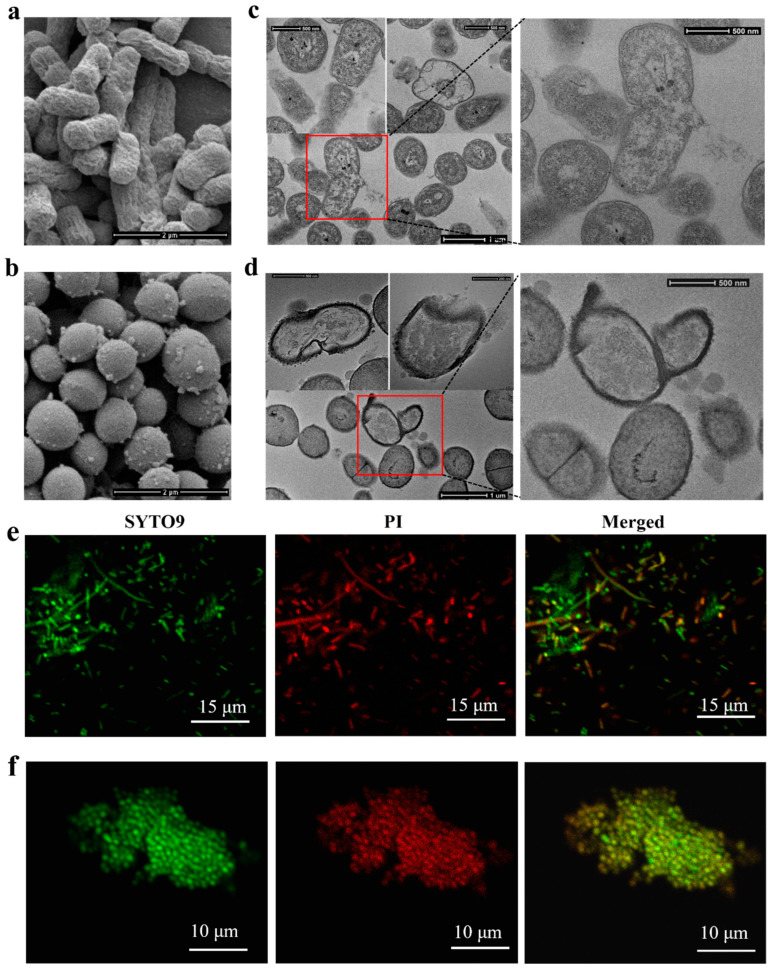
SEM images of *E. coli* ATCC25922 (**a**) and *S. aureus* ATCC25923 (**b**) treated with 2× MIC of C10CL5 for 1 h. TEM images of *E. coli* ATCC25922 (**c**) and *S. aureus* ATCC25923 (**d**) treated with 2× MIC of C10CL5 for 1 h. CLSM images of *E. coli* ATCC25922 (**e**) and *S. aureus* ATCC25923 (**f**) treated with 2× MIC of C10CL5 for 1 h. SEM, TEM and CLSM images of the untreated bacteria are presented in the Supplementary Materials Figure S10.

SEM was used to observe the effect of C10CL5 on the morphology of bacterial cells. The untreated *E. coli* ATCC25922 and *S. aureus* ATCC25923 showed intact, plump and uniform morphologies (Figure S10a,b). However, after treatment with 2× MIC of C10CL5 for 1 h, the surface of *E. coli* became rough, exhibiting obvious wrinkles, shrinkage, and deformation (Figure 6a); and the surface of *S. aureus* became rough, with distinct vesicular protrusions (Figure 6b).

TEM was used to further observe the ultrastructural changes of bacterial cells. The untreated bacteria had intact cell membrane structures and dense cellular contents (Figure S10c,d). In sharp contrast, after treatment with 2× MIC of C10CL5 for 1 h, both *E. coli* and *S. aureus* showed marked cellular vacuolization, diffused cytoplasmic contents, cell membrane dissolution and fragmentation, as well as leakage of cellular contents (Figure 6c,d).

SYTO 9 can penetrate intact or damaged bacterial membranes, staining bacteria green, while PI can only penetrate damaged ones, staining them red [18]. From Figure S10e,f, the untreated bacteria were only stained green by SYTO9, indicating intact bacterial membranes. In contrast, the bacteria treated with C10CL5 exhibited a yellow color (overlap of green and red), indicating that the bacterial membranes were damaged (Figure 6e,f). PI entered the damaged bacterial cells, bound to DNA, and emitted red fluorescence. These observations further confirmed that C10CL5 induced bacterial membrane damage.

**Figure 7:**
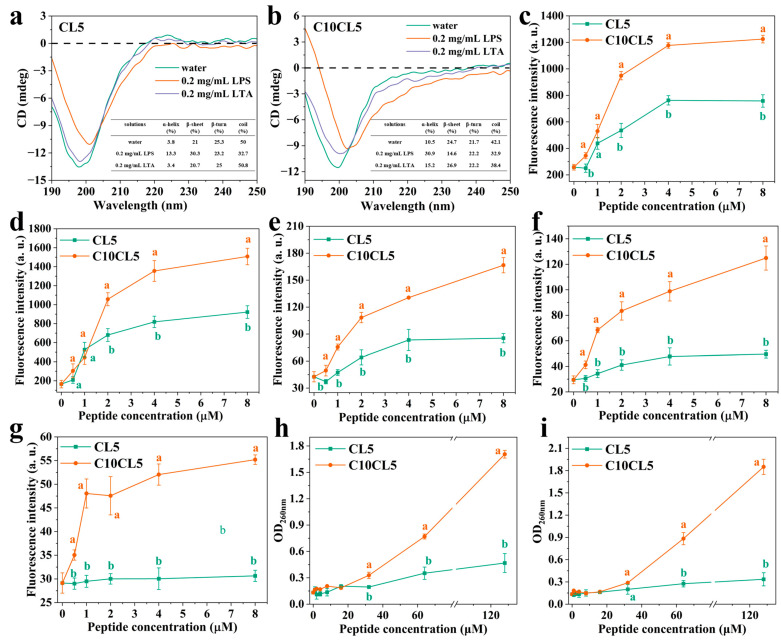
CD spectra of CL5 (**a**) and C10CL5 (**b**); the inset show the proportions of α-helix, β-sheet, β-turn and random coil in the secondary structure. Effect of C10CL5 on the cytoplasmic membrane depolarization of (**c**) *E. coli* ATCC25922 and (**d**) *S. aureus* ATCC25923. Effect of C10CL5 on the outer membrane permeability of *E. coli* ATCC25922 (**e**). Effect of C10CL5 on the inner membrane permeability of *E. coli* ATCC25922 (**f**) and *S. aureus* ATCC2592 (**g**). Effect of C10CL5 on the nucleic acid leakage of *E. coli* ATCC25922 (**h**) and *S. aureus* ATCC2592 (**i**). The data are expressed as the mean ± SD from three independent experiments. In (**c**–**i**): Different lowercase letters of the same color indicate significant differences, *p* < 0.05.

From the CD spectra (Figure 7b), C10CL5 showed a strong negative peak at ~198 nm in deionized water. In LPS solution, the CD spectrum of C10CL5 underwent a red shift, with the negative peak appearing at ~204 nm; while in LTA solution, the negative peak shifted to ~201 nm. These results indicated that the secondary structure of C10CL5 changed when it interacted with bacterial surface components. CDPro analysis results indicated that C10CL5 exhibited higher α-helix proportion than CL5 in LPS and LTA solutions (the inset of Figure 7a,b), suggesting that C10CL5 has a stronger ability to disrupt bacterial membranes than CL5 [19].

The membrane-potential sensitive dye DiSC3(5) was used to evaluate the depolarization effect of C10CL5 on bacterial cytoplasmic membranes [20]. After treating *E. coli* and *S. aureus* with C10CL5, the fluorescence intensity of DiSC3(5) increased rapidly in a concentration-dependent manner (Figure 7c,d), indicating that C10CL5 could induce bacterial cytoplasmic membrane damage, leading to their depolarization. At concentrations greater than 1 μM, C10CL5 exhibited significantly higher depolarization activity than CL5 (*p* < 0.05).

The NPN fluorescent probe was used to detect the effect of C10CL5 on the permeability of bacterial outer membranes. NPN is a hydrophobic fluorescent dye that can only enter bacteria with damaged outer membranes and emit strong fluorescence [21]. As shown in Figure 7e, the NPN fluorescence intensity increased in a concentration-dependent manner as the concentration of C10CL5 increased, and was significantly higher than that induced by CL5 at the same concentration (*p* < 0.05). This indicated that C10CL5 exhibited a stronger ability to disrupt bacterial outer membranes than CL5 (*p* < 0.05).

The PI fluorescent probe was used to detect the effect of C10CL5 on the permeability of bacterial inner membranes. PI can enter bacteria with damaged inner membranes and bind to DNA to emit red fluorescence [22]. As shown in Figure 7f,g, the PI fluorescence intensity increased in a concentration-dependent manner as the concentration of C10CL5 increased, and was significantly higher than that induced by CL5 at the same concentration (*p* < 0.05). This indicated that C10CL5 exhibited a stronger ability to disrupt the inner membrane integrity of bacteria than CL5 (*p* < 0.05).

At concentrations of 1–16 μM, the level of nucleic acid leakage induced by C10CL5 showed no significant difference from that induced by CL5 at the same concentrations. However, as the concentration of C10CL5 increased, the nucleic acid leakage of *E. coli* ATCC25922 and *S. aureus* ATCC25923 induced by C10CL5 was significantly higher than that induced by CL5 at the same concentration (*p* < 0.05). At 128 μM, the nucleic acid leakage of *E. coli* induced by C10CL5 was more than twice that induced by CL5, and the nucleic acid leakage of *S. aureus* induced by C10CL5 was more than four times that induced by CL5 (Figure 7h,i).

### 2.7. Effects of Lipopeptides on Bacterial Genomic DNA

**Figure 8:**
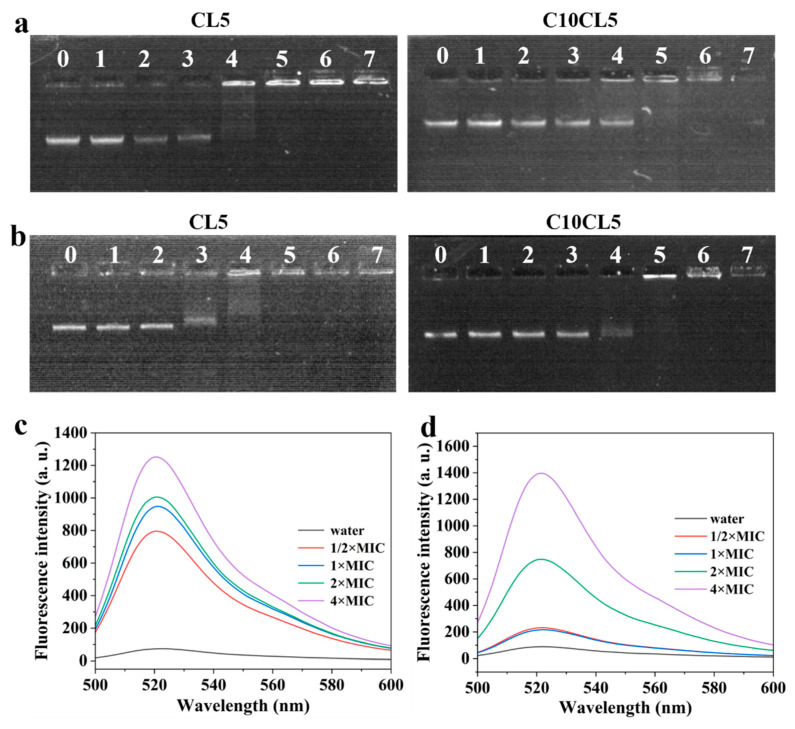
Agarose gel retardation assay of CL5 and C10CL5 with *E. coli* ATCC25922 (**a**) and *S. aureus* ATCC25923 (**b**) genomic DNA. Lanes 0–7 represent peptide concentrations of 0, 2, 4, 8, 16, 32, 64 and 128 μM, respectively. Effect of C10CL5 on cellular ROS production of *E. coli* ATCC25922 (**c**) and *S. aureus* ATCC25923 (**d**).

Agarose gel electrophoresis was used to investigate the binding ability of C10CL5 to bacterial genomic DNA. For *E. coli* genomic DNA, the migration rate in the gel was similar to that of the control group when the C10CL5 concentration was 2–8 μM. When C10CL5 concentration was increased to 16 μM, the DNA migration rate decreased slightly; further increasing to 32 μM resulted in the disappearance of the DNA band, which was retained in the sample wells (Figure 8a). The binding of C10CL5 to *S. aureus* genomic DNA exhibited a similar pattern as described above (Figure 8a). Notably, the parental peptide CL5 at 16 μM could retain the genomic DNA of *E. coli* and *S. aureus* in the sample wells, indicating that CL5 had a stronger DNA-binding capacity than C10CL5. This might be attributed to the higher positive charge of CL5 (CL5: +8, C10CL5: +7), which enhanced the electrostatic interactions between CL5 and negatively charged genomic DNA, thereby resulting in stronger binding affinity.

### 2.8. Effects of Lipopeptides on Bacterial Reactive Oxygen Species

The ROS Assay Kit was used to detect the effect of C10CL5 on the production of cellular ROS. DCFH-DA is an oxidation-sensitive probe that is non-fluorescent itself, but it can penetrate cell membranes and be hydrolyzed into DCFH by cellular esterases. Subsequently, DCFH is further oxidized by ROS to generate the fluorescent product DCF [23]. The results (Figure 8c,d) showed that the fluorescence intensity of DCF increased with the increase in C10CL5 concentration, indicating that C10CL5 promoted the accumulation of cellular ROS in bacteria.

## 3. Discussion

The global spread of drug-resistant bacteria has become a major public health challenge, and the efficacy of traditional antibiotics has declined sharply due to the continuous emergence of drug-resistant strains, making the development of novel antimicrobial agents an urgent research priority [24]. AMPs possess distinct advantages such as wide sources, broad-spectrum antimicrobial activity, and low tendency to induce bacterial resistance, and thus have great application potential in the food industry, feed production, and biomedicine [25]. However, AMPs generally suffer from inherent drawbacks such as low antimicrobial activity, high cytotoxicity and poor stability, which have severely limited their practical applications. In this study, we designed a series of lipopeptides by conjugating fatty acid chains of varying lengths to the N-terminus of the LPS-targeting AMP CL5, with the aim of screening out highly effective and low-toxicity antimicrobial alternatives.

The retention time of peptides on an RP-HPLC column can reflect their hydrophobicity; under the same elution conditions, a longer retention time indicates a greater hydrophobicity of the peptide [26]. In this work, fatty acid chain modification significantly increased the hydrophobicity of lipopeptides, and the hydrophobicity was positively correlated with the length of the fatty acid chain, which was consistent with the results of a previous study [14].

Regarding the effect of the self-assembly of AMPs on their activity, some studies have shown that self-assembly can enhance the antimicrobial activity of AMPs [27,28], while others demonstrate that this process impedes the interaction between AMPs and bacterial membranes, thus reducing antimicrobial activity [23,29]. Our previous study has shown that the self-assembling C12-CL5 had high antimicrobial activity [17], and herein, we further investigated the self-assembly properties of other fatty acid-modified peptides, so as to provide a more comprehensive explanation for their antimicrobial activity.

The hydrophobic fluorescent probe 1,8-ANS is a classic tool for detecting peptide self-assembly: it shows weak fluorescence in aqueous solution, and its fluorescence intensity increases significantly with a blue shift when bound to the hydrophobic regions of self-assembled peptides [30]. Our results showed that lipopeptides modified with short fatty acid chains (C2CL5–C6CL5) had no obvious self-assembly ability in the concentration range of 1–256 μM, while lipopeptides modified with long fatty acid chains (C8CL5–C18CL5) exhibited significant self-assembly ability. The self-assembly ability was enhanced with the elongation of the fatty acid chain, which was reflected by the gradual decrease in the CAC values.

Amphiphilic peptides with self-assembling ability can assemble into various nanostructures (e.g., spherical, lamellar, rod-like and fibrous nanostructures) in aqueous solution [31,32]. C8CL5–C18CL5 could self-assemble into positively charged spherical-like nanostructures in aqueous solution. This property facilitated the electrostatic binding of lipopeptides to negatively charged bacterial membranes, laying the structural foundation for their antimicrobial activity. MD simulations further confirmed the self-assembly potential of lipopeptides modified with longer fatty acid chains, and revealed that van der Waals forces between fatty acid chains were the core driving force for lipopeptide self-assembly. Specifically, lipopeptides C8CL5–C18CL5 were dispersed as monomers in aqueous solutions at low concentrations. When their concentration reached the CACs, the N-terminal fatty acid chains aggregated, driven by van der Waals forces, thus reducing contact with polar water molecules. Meanwhile, the hydrophilic peptide chains extended and arranged on the outer layer of the self-assembled structures. The self-assembled nanostructures carried positive charges due to the presence of basic amino acids (lysine and arginine) in the peptide chain, which was consistent with the results of zeta potential measurements. The electrostatic repulsion between positive charges prevented excessive aggregation of the assemblies, enabling their stable dispersion in aqueous solution.

Antimicrobial assay results demonstrated that lipopeptides modified with shorter fatty acid chains (2–6 carbon atoms) exhibited weaker activity than the parental peptide CL5. This may be because N-terminal fatty acid modification reduced one positive charge of the peptide, thus weakening the electrostatic interaction between the peptide and the negatively charged bacterial membrane; meanwhile, the slight increase in hydrophobicity cannot offset the negative impact of charge reduction, ultimately leading to decreased activity of C2CL5–C6CL5. In contrast, the moderate hydrophobicity of lipopeptides C8CL5–C14CL5 facilitated the balance of structural parameters of AMPs [33]. Furthermore, the self-assembly of this series increased the local density of hydrophobic and positively charged amino acid residues, enhancing the interactions of lipopeptides with bacterial membranes [34]. However, the excessive hydrophobicity of lipopeptides C16CL5 and C18CL5 disrupted the structural parameter balance, ultimately resulting in the reduction of their activity. Consistent with the trends reported in existing literature, the GM values of the lipopeptides in this study exhibited an approximately “U”-shaped relationship with fatty acid chain length [14,23]. What differs, however, was that the lipopeptides demonstrating superior antimicrobial activity in this study had a 10-carbon fatty acid chain, a finding was not entirely consistent with other relevant research reports. For instance, the study by zhong et al. showed that lipopeptides Cn-W1R2(RWR) with a 14-carbon fatty acid chain exhibited the strongest antimicrobial activity [23]. This discrepancy was speculated to stem from the differences in the amino acid sequences and structural characteristics of the parental peptides. Furthermore, C8CL5 and C10CL5 could rapidly eradicate *E. coli* and *S. aureus*, and this property effectively reduced the probability of bacterial resistance development, which was a favorable characteristic for their clinical application.

Cytotoxicity is a major obstacle limiting the application of AMPs. In this study, the introduction of medium-to-long fatty acid chains (8–14 carbon atoms) at the N-terminus of CL5 not only significantly enhanced the antimicrobial activity of the lipopeptide but also induced a certain degree of cytotoxicity to mammalian cells. Although the more active lipopeptides C8CL5–C12CL5 exhibited certain cytotoxicity at high concentrations, their cytotoxicity remained relatively low at concentrations corresponding to the GM_all_ values. The SI calculation showed that C8CL5–C12CL5 had relatively high SI_all_ values of 12.26, 8.14, and 7.48, respectively, indicating their superior safety profiles [35]. Owing to the differences in cell membrane composition and structure between bacteria and mammalian cells, fatty acid chain modification of AMPs could induce disproportionate changes in their antimicrobial activity and cytotoxicity. In this work, C8CL5–C12CL5 exhibited significantly greater enhancement in antimicrobial activity than that in cytotoxicity, thus demonstrating superior antimicrobial selectivity against bacteria.

Poor stability is another key bottleneck hindering the pharmaceutical translation and large-scale application of AMPs. C10CL5 exhibited excellent thermal, pH, and salt stability, which was consistent with the stability performance of other reported lipopeptides. Previous studies have indicated that the appropriate enhancement of hydrophobicity and positive charge of AMPs can improve their salt tolerance [36]. C10CL5 monomers possessed high hydrophobicity and carried positive charges, and the formation of self-assemblies at concentrations above the CAC further increased the local density of hydrophobic and positively charged amino acids. Thus, C10CL5 exhibited good antimicrobial activity in saline environments. AMPs are prone to being hydrolyzed into shorter peptide fragments or amino acids by proteases, resulting in reduced or lost activity. C10CL5 contains multiple lysine residues, and the carboxyl terminus of lysine serves as a cleavage site for trypsin [37]. Thus, C10CL5 showed poor resistance to trypsin and was readily hydrolyzed, resulting in loss of activity, a characteristic similar to that of CL5. This enzymatic hydrolysis property also indirectly reflected the relative safety of C8CL5 and C10CL5—they could be degraded by trypsin in vivo, thereby reducing their cytotoxicity and impact on gut microbiota [38].

The antimicrobial mechanism of AMPs is complex and diverse, mainly including disrupting bacterial cell membranes, inhibiting protein/nucleic acid synthesis, suppressing the activity of intracellular enzymes, and interacting with intracellular substances [39,40]. Taking lipopeptide C10CL5, which possessed high antimicrobial activity, low cytotoxicity and excellent stability, as a representative, this study further investigated its antimicrobial mechanism. A variety of experimental methods including SEM, TEM, CLSM, DiSC3(5) cytoplasmic membrane depolarization assay, NPN outer membrane permeability assay, PI inner membrane permeability assay and nucleic acid leakage assay confirmed that C10CL5 could significantly induce the depolarization of bacterial cytoplasmic membranes, disrupt the integrity of bacterial outer and inner membranes in a concentration-dependent manner, and cause the leakage of cellular nucleic acids. In addition to the membrane-damaging mechanism, C10CL5 could also interact with bacterial genomic DNA to interfere with the normal physiological activities of bacteria, and promote the production of cellular ROS to cause oxidative damage to bacterial cells [41].

We speculate that the three antimicrobial mechanisms (membrane disruption, DNA binding and ROS accumulation) exhibited sequential and synergistic relationships. The lipopeptides first destroyed the structural integrity of bacterial cell membranes, then entered the cells and bound to genomic DNA to interfere with its physiological functions. Meanwhile, the above processes further promoted the accumulation of ROS, causing oxidative damage to bacteria. These three mechanisms acted in a cascading and synergistic manner, ultimately achieving potent antimicrobial activity. The multi-target antimicrobial mechanism of C10CL5 was conducive to reducing the probability of bacterial resistance, which was an important advantage compared with traditional antibiotics with a single mechanism of action.

## 4. Materials and Methods

### 4.1. Chemicals and Reagents

N-phenyl-1-naphthylamine (NPN, 98%), 3,3’-dipropylthiadicarbocyanine iodide (DISC3(5), ≥98%), Polymyxin B (PMB, USP, ≥6000 USP units/mg), Nisin Z (≥1000 IU/mg) and 8-anilino-1-naphthalenesulfonic acid (1,8-ANS, 96%) were obtained from Aladdin Biochemical Technology Co., Ltd. (Shanghai, China). Propidium iodide (PI, Biotech, 94%) was purchased from Macklin Biochemical Co., Ltd. (Shanghai, China). SYTO9/PI Live/Dead bacterial double stain kit was purchased from Fushen Biotechnology Co., Ltd. (Shanghai, China). Bacterial genomic DNA isolation kit was purchased from Sangon Biotech Co., Ltd. (Shanghai, China). The reactive oxygen species assay kit was purchased from Beyotime Biotechnology Co., Ltd. (Shanghai, China). All other reagents were commercially available and of analytical grade.

### 4.2. Bacterial Strains and Growth Conditions

The bacterial strains used in this study included *E. coli* (ATCC25922, O157:H7, CMCC44102, ATCC8739), *P. aeruginosa* (ATCC27853, ATCC9027, CMCC10104), *S. typhimurium* ATCC14028, *S. enteritidis* CVCC3375, *S. sonnei* ATCC25931, *S. flexneri* CMCC51572, *S. putrefaciens* BNCC337021, *S.paratyphi* CMCC50094, *P. fluorescens* BNCC336632, *S. aureus* (ATCC43300, ATCC25923), *B. subtilis* ATCC6633, *L. monocytogenes* ATCC19115, *B. licheniformis* CGMCC2876. The bacteria used in this study were cultured in Luria–Bertani (LB) liquid medium at 37 °C with constant shaking at 150 rpm for subsequent experiments.

### 4.3. Synthesis and Characterization of Lipopeptides

All lipopeptides in this study were custom-synthesized by GL Biochem Co., Ltd. (Shanghai, China) using the Fmoc solid-phase peptide synthesis (SPPS) method. Prior to use, these lipopeptides were characterized in our laboratory. ESI-MS (Thermo Fisher, Waltham, MA, USA) was used to verify the molecular weight of the lipopeptides, and RP-HPLC (Agilent, Santa Clara, CA, USA) with an Agilent Zorbax SB column (9.4 mm × 250 mm, 5 µm particle size) was used to determine the purity of the lipopeptides.

### 4.4. Self-Assembly of Lipopeptides

The hydrophobic fluorescent probe 1,8-ANS was used to detect the self-assembly ability of lipopeptides in aqueous solution. CD spectroscopy (JOSCO, Tokyo, Japan) was used to analyze the secondary structure of the lipopeptides. AFM (Oxford, Oxford, UK) in tapping mode was used to observe the morphology of lipopeptide self-assemblies. A zeta potential analyzer (Malvern, Malvern, UK) was used to measure the zeta potential of the lipopeptides.

### 4.5. Molecular Dynamics (MD) Simulations

Firstly, the 3D structure of peptide sequence H-KWKLFKKIRKVRGPP-OH was constructed using Alphafold2 [42]. Fatty acid structures obtained from PubChem (https://pubchem.ncbi.nlm.nih.gov/, accessed on 23 January 2024) were optimized using Avogadro software 2.0.0 (force field: MMFF94s) [43], and then optimized at the B97-3c level using ORCA with water as the SMD solvent [44]. The lipopeptide models were constructed using the GaussView 6.0 program. The above optimized fatty acid structures were attached to the N-terminus of H-KWKLFKKIRKVRGPP-OH, and the C-terminus of H-KWKLFKKIRKVRGPP-OH was amidated. The GAFF topological parameters of the lipopeptides were constructed by Sobtop tool (Tian Lu, Sobtop, Version [1.0(dev3.1)], http://sobereva.com/soft/Sobtop, accessed on 13 December 2023).

All-atom MD simulations were performed using the GROMACS 21.4 package (force field: amber14sb_parmbsc1) [45]. In the initial configuration, each type of modified lipopeptide was present in a quantity of 50, and they were randomly distributed within a 12 nm × 12 nm × 12 nm cubic box of the TIP3P water model, and then counterions were added to neutralize the charge of the system. The energy of the system was reduced to 100.0 kJ/mol/nm using the steepest descent method and conjugate gradient optimization. Then, the system was pre-balanced using canonical ensemble (NVT, 1 ns) and isothermal–isobaric (NPT, 1 ns). Finally, all restrictions were lifted and 500 ns MD simulations were performed [46]. Considering that our goal is to conduct a qualitative comparison of co-assemblies, only one molecular dynamics simulation is performed for each group. Detailed simulation parameters and files can be downloaded at https://zenodo.org/records/20231862 (accessed on 16 May 2026).

### 4.6. Independent Gradient Model (IGM) Analysis

IGM is a widely adopted method for studying weak interactions, which can detect hydrogen bonding and van der Waals interactions between fragments [47]. Therefore, the IGM method was used to analyze the weak interaction between lipopeptides and lipopeptides. The IGM was calculated by the Multiwfn 3.8 (dev) program [48], and the visual analysis of the weak interaction was performed using VMD 1.9.3 software [49]. In the IGM analysis, *δg* is a three-dimensional real-space function related to all the interatomic interactions in the system. The stronger the interatomic interactions, the larger the *δg* in the interatomic interaction region. *δg* can be divided into *δg^intra^* and *δg^inter^*, which represent intra-fragment interactions and inter-fragment interactions, respectively. And for the calculation of *δg^inter^* between multiple fragments according to the following Equations (1)–(3), of which A indicates the fragment number. In this study, we selected representative clusters from molecular dynamics simulations, and used the IGM method to visualize the non-covalent interactions between different lipopeptides within these clusters.

(1) $$g^{inter}(r)=\left|\left.\sum_{A}\sum_{i\in A}\nabla \rho_{i}(r)\right|\right.$$

(2) $$g^{IGM,inter}(r)=\left|\left.\sum_{A}\mathrm{abs}\left[\sum_{i\in A}\nabla \rho_{i}(r)\right]\right|\right.$$

(3) $$\delta g^{inter}(r)=g^{IGM,inter}(r)-g^{inter}(r)$$

### 4.7. Determination of Minimum Inhibitory Concentration (MIC) and Minimum Bactericidal Concentration (MBC)

The minimum inhibitory concentration (MIC) and minimum bactericidal concentration (MBC) of lipopeptides were determined to evaluate their antimicrobial activity, and the experimental procedures were carried out according to our previous report [14]. PMB and Nisin Z were used as positive controls in the assay.

### 4.8. Bacterial Growth Curve

The bacterial suspension was mixed with an equal volume of lipopeptide at different concentrations in a 96-well plate and incubated at 37 °C. At preset time points, the optical density (OD) values at 630 nm were measured using a MB-580 microplate reader (Huisong technology, Shenzhen, China), and the OD_630_ values of the bacterial suspension were plotted against incubation times.

### 4.9. Time-Killing Kinetics Assay

The time-killing kinetics of lipopeptides at 1×, 2×, and 4× MIC against *E. coli* ATCC25922 and *S. aureus* ATCC25923 were measured within 8 h, and the number of viable bacteria at different time points was counted to evaluate the bactericidal rate of the lipopeptide.

### 4.10. Cytotoxicity Assay

The MTT method was used to evaluate the cytotoxicity of lipopeptides against HEK-293 cells (purchased from Pricella Biotechnology Co., Ltd., Wuhan, China), and the IC_50_ was calculated based on the experimental results. The SI was further calculated using the formula: SI = IC_50_/GM (GM is the geometric mean of the MIC values of lipopeptides against bacteria). Detailed experimental methods were described in our previous report [14].

### 4.11. Thermal, pH, Salt and Protease Stability

The thermal stability of C10CL5 was evaluated by treating the peptide at 60, 80, 100 and 121 °C for 1 h. The pH stability was determined by incubating the peptide in buffer solutions with pH values of 2, 4, 6, 8, 10 and 12 for 3 h. The salt stability was evaluated by determining the MIC of C10CL5 in the presence of different physiological salts (150 mM NaCl, 4.5 mM KCl, 2.5 mM CaCl_2_, 1 mM MgCl_2_, 4 μM FeCl_3_, 8 μM ZnCl_2_, 6 μM NH_4_Cl). The MICs against *E. coli* ATCC25922 and *S. aureus* ATCC25923 of all treated samples were determined by the two-fold dilution method.

The protease stability was tested by mixing C10CL5 (4× MIC) with equal volumes of trypsin solutions (0.02, 0.2 and 2 mg/mL) and incubating at 37 °C for 4 h; the mixture was then heated at 80 °C for 10 min to inactivate trypsin, and the antimicrobial activity was determined. The bacterial growth rate was calculated using the formula:

Bacterial growth (%) = (OD_sample_ − OD_blank_)/(OD_control_ − OD_blank_) × 100%

### 4.12. SEM and TEM Imaging

The bacterial suspension (OD_600_ = 0.2–0.5, in PBS solution) was treated with 2× MIC of lipopeptides for 1 h at 37 °C under shaking (150 rpm). The untreated bacteria were used as control. The bacteria were fixed, washed, dehydrated, replaced, lyophilized, and gold-sputtered before being observed under a Quanta 250FEG SEM (Thermo Fisher, Waltham, MA, USA). Additionally, the bacteria were fixed, washed, dehydrated, embedded, sectioned, and stained before being observed using a FEI TalosF200S TEM (Thermo Fisher, Waltham, MA, USA). Detailed experimental methods were described in our previous report [14].

### 4.13. CLSM Observation

The bacterial suspension (1 × 10^5^–5 × 10^5^ cfu/mL, in saline) was treated with 2× MIC of lipopeptides for 1 h at 37 °C under shaking (150 rpm). Subsequently, the bacteria were washed, re-suspended in saline, and stained with the dye mixture (SYTO9:PI = 1:1, *v*:*v*) at a ratio of 1 mL:3 μL. After incubation in the dark for 15 min, the bacteria were washed, re-suspended with saline, and observed under an Olympus FV3000 CLSM (Olympus, Tokyo, Japan).

### 4.14. CD Spectroscopy

The secondary structures of the lipopeptides in different environments (deionized water, 0.2 mg/mL LPS, 0.2 mg/mL LTA) were measured on a J-1500 CD spectrometer (Jasco, Tokyo, Japan). The final concentration of the peptide was 50 μM, and the CD spectra were recorded in the wavelength range of 190–260 nm at room temperature with a scanning speed of 100 nm/min. Each sample was scanned three times. Baseline calibration was performed with peptide-free solutions.

### 4.15. Cytoplasmic Membrane Depolarization Assay

The membrane-potential sensitive fluorescent dye DiSC3(5) was used to determine the depolarization activity of lipopeptides. The bacterial suspension (OD_600_ = 0.05, in HEPES buffer) was incubated with DiSC3(5) (final concentration 2 μM) at 37 °C in the dark until the fluorescence intensity at 670 nm (excitation wavelength 622 nm) reached the minimum value. Different concentrations of lipopeptides were then added to the bacterial suspension, and after incubation at 37 °C for 1 h, the fluorescence intensity at 670 nm was measured to evaluate the membrane depolarization effect.

### 4.16. Outer/Inner Membrane Permeability Assay

The NPN fluorescent probe was used to detect the effect of lipopeptides on the outer membrane permeability of *E. coli*. NPN (final concentration 10 μM) and lipopeptides (final concentration 0–32 μM) were added to the bacterial suspension (OD_600_ = 0.2–0.5, in HEPES buffer), and after incubation at 37 °C for 1 h, the fluorescence intensity at 420 nm (excitation wavelength 350 nm) was measured. The PI fluorescent probe was used to detect the inner membrane permeability of bacteria. PI (final concentration 10 μM) and lipopeptides (final concentration 0–32 μM) were added to the bacterial suspension (OD_600_ = 0.2–0.5, in 10 mM PBS), and after incubation at 37 °C for 1 h, the fluorescence intensity at 617 nm (excitation wavelength 535 nm) was measured.

### 4.17. DNA Gel Electrophoresis

Bacterial genomic DNA was extracted using a bacterial genomic DNA isolation kit according to the manufacturer’s instructions, and the OD_260_/OD_280_ ratio was used to evaluate the purity of the DNA. 5 μL of lipopeptide solution was mixed with 5 μL of bacterial genomic DNA and incubated at room temperature for 1 h. Subsequently, 1 μL of 6× loading buffer was added to the mixture, and agarose gel electrophoresis (containing 0.5 μg/mL ethidium bromide) was performed to analyze the binding ability of lipopeptides to genomic DNA.

### 4.18. ROS Assay

The lipopeptides of different concentrations were mixed with an equal volume of bacterial suspension (OD_600_ = 0.5–0.8, in PBS solution), and DCFH-DA was added to a final concentration of 10 μM. After incubation at 37 °C for 1 h, the fluorescence intensity at 525 nm (excitation wavelength 488 nm) was measured using an RF-6000 fluorescence spectrophotometer (SHIMADZU, Kyoto, Japan) to evaluate the level of cellular ROS in bacteria.

### 4.19. Statistical Analysis

The one-way analysis of variance (ANOVA) and Duncan test were used for significance analysis, and *p* < 0.05 was considered to be statistically significant.

## 5. Conclusions

In this study, a series of lipopeptides were designed by conjugating fatty acid chains of varying lengths to the N-terminus of the LPS-targeting AMP CL5. The correlations among fatty acid chain length, self-assembly behavior, and antimicrobial activity were investigated, and the antimicrobial mechanism of the lipopeptides was explored. The results indicated that lipopeptides C8CL5–C18CL5 could self-assemble into nanostructures with fatty acid chains as the inner core and hydrophilic peptide chains as the outer shell in aqueous solution, and the self-assembly ability of lipopeptides was enhanced with the elongation of the fatty acid chain. Hydrophobic interactions between fatty acid chains were the primary driving force for lipopeptide self-assembly. The GM values of MICs of the lipopeptides exhibited an approximate “U”-shaped relationship with fatty acid chain length. Among these lipopeptides, C8CL5–C12CL5 exhibited potent broad-spectrum antimicrobial activity, which was significantly superior to that of the parental peptide CL5. In addition, the lipopeptides possessed rapid time-killing kinetics, along with favorable thermal, pH, and salt stability, but they were sensitive to trypsin. Mechanistic studies revealed that the representative lipopeptide C10CL5 could induce the depolarization of bacterial cytoplasmic membranes, disrupt the integrity of bacterial outer and inner membranes, leading to the leakage of intracellular contents. Furthermore, C10CL5 was also found to interact with bacterial genomic DNA and promote the generation of cellular ROS. This study provides valuable insights for the rational design of highly active lipopeptides.

## Data Availability

Data are available upon request.

## Supplementary Materials

The following supporting information can be downloaded at: https://www.mdpi.com/article/10.3390/antibiotics15050518/s1, Figure S1. Chemical structure of the lipopeptides CnCL5. Figure S2. ESI-MS (left) and RP-HPLC (right) spectra of the lipopeptides. Figure S3. The fluorescence intensity of 1,8-ANS against the logarithmic values of lipopeptide concentrations. Figure S4. (a) CD spectra of C8CL5−C18CL5 at 100µM; the inset shows the CD spectra of C8CL5−C18CL5 at 6 µM. (b) Estimation of secondary structure proportions of C8CL5−C18CL5 at 100 μM using CDpro. Figure S5. AFM images of (a) C2CL5, (b) C4CL5 and (c) C6CL5. Figure S6. Zeta potentials of C8CL5−C18CL5. The measurements were repeated three times, and the data are expressed as the mean±SD. Figure S7. Effect of different concentrations of C10CL5 on the growth curves of *P. aeruginosa* ATCC27853 (a), *S. typhimurium* ATCC14028 (b), *S. aureus* ATCC43300 (c), *B. subtilis* ATCC6633 (d). The measurements were repeated three times, and the data are expressed as the mean±SD. Figure S8. Effects of lipopeptides at different concentrations on the viability of HEK-293 cells. The measurements were repeated three times, and the data are expressed as the mean±SD. Figure S9. The IC50 values of the lipopeptides toward HEK-293 cells. Figure S10. SEM (a, b), TEM (c, d) and CLSM (e, f) images of the untreated *E. coli* ATCC25922 (a, c, e) and *S. aureus* ATCC25923 (b, d, f). Table S1. Amino acid sequences and physicochemical properties of the lipopeptides. Table S2. The MIC and GM values of the lipopeptides.

## Abbreviations

The following abbreviations are used in this manuscript:

ESI-MS	Electrospray ionization mass spectrometry
RP-HPLC	Reversed-phase high-performance liquid chromatography
AFM	Atomic force microscopy
SEM	Scanning electron microscopy
TEM	Transmission electron microscopy
CLSM	Confocal laser scanning microscopy
MD	Molecular dynamics
IGM	Independent gradient model
CD	Circular dichroism
OM	Outer membrane
IM	Inner membrane
ROS	Reactive oxygen species
CAC	Critical aggregation concentration
MIC	minimum inhibitory concentration
IC_50_	half maximal inhibitory concentration
SI	Selectivity index
