# Supplementary material for: Effect of Fatty Acid Chain Modification on the Self-Assembly Behavior and Antimicrobial Activity of Antimicrobial Peptides
Source: Antibiotics (Basel). 2026 May 20;15(5):518. doi: 10.3390/antibiotics15050518 (PMC13203949; doi:10.3390/antibiotics15050518)
Supplement: Supplementary file 1 [file antibiotics-15-00518-s001.zip › antibiotics-4313404-supplementary.pdf]

Supplementary material

Effect of fatty acid chain modification on the self-assembly behavior and antimicrobial activity of antimicrobial peptides

Hongyan Yang[a], Meiqian Luo[a], Yutao Min[a], Yehuan Zheng[b], Yanhua Xu[a], Bingchao Duan[a], Fei Pan[c, *], Kui Lu[a,d*]

[a] School of Food and Health Engineering, Zhengzhou University of Technology, Zhengzhou, 450044, China.

[b] Molecular Diagnostics Department, R&D Center, Autobio Diagnostics Co., Ltd, Zhengzhou, 450016, China.

[c] State Key Laboratory of Resource Insects, Institute of Apicultural Research, Chinese Academy of Agricultural Sciences, Beijing, 100093, China.

[d] College of Food Science and Engineering, Henan University of Technology, Zhengzhou, 450001, China.

Corresponding authors:

Fei Pan: yunitcon@yeah.net

Kui Lu: lukui126@126.com

1. Supplementary Tables

Table S1. Amino acid sequences and physicochemical properties of the lipopeptides.

Peptide	Sequence	Theoretical MW (Da)	Measured MW (Da) [a]	Purity (%) [b]	Net charge [c]	Rt (min) [d]
CL5	KWKLFKKILPRKVRGPP-NH_2	2090.64	2089.2	96.858	+8	8.485
C2CL5	C2-KWKLFKKILPRKVRGPP-NH_2	2132.69	2131.0	96.856	+7	10.646
C4CL5	C4-KWKLFKKILPRKVRGPP-NH_2	2160.75	2159.0	96.148	+7	11.069
C6CL5	C6-KWKLFKKILPRKVRGPP-NH_2	2188.80	2186.8	98.237	+7	16.781
C8CL5	C8-KWKLFKKILPRKVRGPP-NH_2	2216.85	2215.3	98.305	+7	16.912
C10CL5	C10-KWKLFKKILPRKVRGPP-NH_2	2244.90	2243.2	98.173	+7	20.138
C12CL5	C12-KWKLFKKILPRKVRGPP-NH_2	2272.96	2270.7	96.241	+7	23.592
C14CL5	C14-KWKLFKKILPRKVRGPP-NH_2	2301.01	2298.7	97.543	+7	26.185
C16CL5	C16-KWKLFKKILPRKVRGPP-NH_2	2329.07	2327.7	98.006	+7	30.317
C18CL5	C18-KWKLFKKILPRKVRGPP-NH_2	2357.12	2354.7	98.569	+7	35.262

[a] Measured MW was determined by ESI-MS. [b] Purity was determined by RP-HPLC. [c] Net charge at pH 7. [d] Retention time (Rt) was determined by RP-HPLC on a C18 column.

Table S2. The MIC and GM values of the lipopeptides.

Bacteria	MICs (μM) [a]											
	CL5	C2CL5	C4CL5	C6CL5	C8CL5	C10CL5	C12CL5	C14CL5	C16CL5	C18CL5	PMB	Nisin Z
Gram-negative bacteria												
E. coli ATCC25922	32	128	128	64	16	4	8	16	>128	>128	1	>128
E. coli O157:H7	8	128	64	32	8	4	4	8	128	>128	1	>128
E. coli CMCC44102	32	32	32	16	4	4	8	16	128	>128	1	>128
E. coli ATCC8739	128	>128	128	64	8	4	8	8	64	64	2	>128
P. aeruginosa ATCC27853	8	>128	>128	64	16	4	8	32	>128	>128	2	>128
P. aeruginosa ATCC9027	32	>128	>128	128	4	4	8	16	64	128	2	>128
P. aeruginosa CMCC10104	32	>128	>128	>128	8	8	8	16	64	128	2	>128
P. fluorescens BNCC336632	8	8	8	4	4	4	8	32	>128	>128	1	>128
S. typhimurium ATCC14028	16	>128	>128	128	16	16	16	64	>128	>128	2	>128
S. enteritidis CVCC3375	8	>128	>128	>128	16	8	16	32	>128	>128	32	>128
S. sonnei ATCC25931	16	32	32	16	4	4	4	8	32	>128	1	>128
S. flexneri CMCC51572	32	128	128	32	2	4	4	4	32	64	1	>128
S. putrefaciens BNCC337021	4	8	4	2	2	4	4	4	8	16	1	>128
S. paratyphi CMCC50094	128	>128	>128	>128	32	8	16	32	>128	>128	2	>128
Gram-positive bacteria												
S. aureus ATCC43300	>128	>128	>128	>128	16	8	32	128	>128	>128	64	64
S. aureus ATCC25923	>128	>128	>128	>128	8	8	16	64	>128	>128	32	128
B. subtilis ATCC6633	>128	>128	>128	>128	2	4	8	32	>128	>128	128	64
L. Monocytogenes ATCC19115	64	>128	>128	32	2	4	4	4	32	128	32	64
B. licheniformis CGMCC2876	>128	32	32	8	2	4	4	8	64	64	128	64
GM_{G-} (μM) [b]	20.49	99.93	86.14	45.25	7.25	5.12	7.61	15.23	99.93	156.03	1.72	256
GM_{G+} (μM) [c]	194.01	168.90	168.90	84.45	4.00	5.28	9.19	24.25	128	168.90	64.00	73.52
GM_{all} (μM) [d]	37.03	114.73	102.84	53.33	6.20	5.16	8.00	17.21	106.66	159.32	4.46	184.35

[a] The MIC values were determined in triplicate with identical results (SD = 0).

[b] Geometric mean (GM_{G-}) of the MIC values of the lipopeptides against Gram-negative bacteria.

[c] Geometric mean (GM_{G+}) of the MIC values of the lipopeptides against Gram-positive bacteria.

When no detectable antimicrobial activity was observed at 128 μM, a value of 256 μM was used to calculate GM_{G-} and GM_{G+}.

[d] Geometric mean (GM_{all}) of the MIC values of the lipopeptides against all the bacteria tested.

2. Supplementary Figures

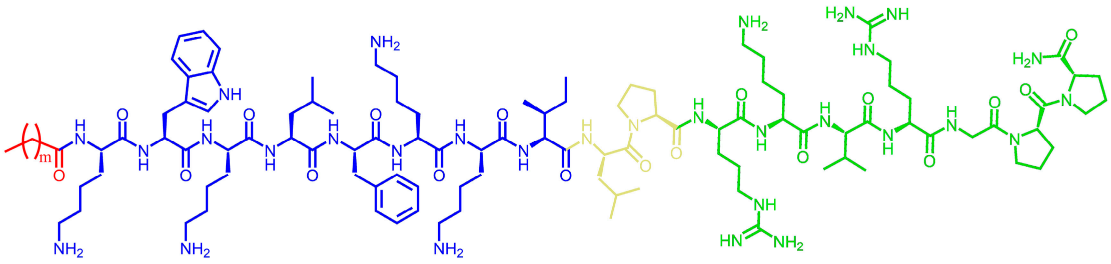

m=0, 2, 4, 6, 8, 10, 12, 14, 16

CnCL5 (n=2, 4, 6, 8, 10, 12, 14, 16, 18)

Figure S1. Chemical structure of the lipopeptides CnCL5.

CL5

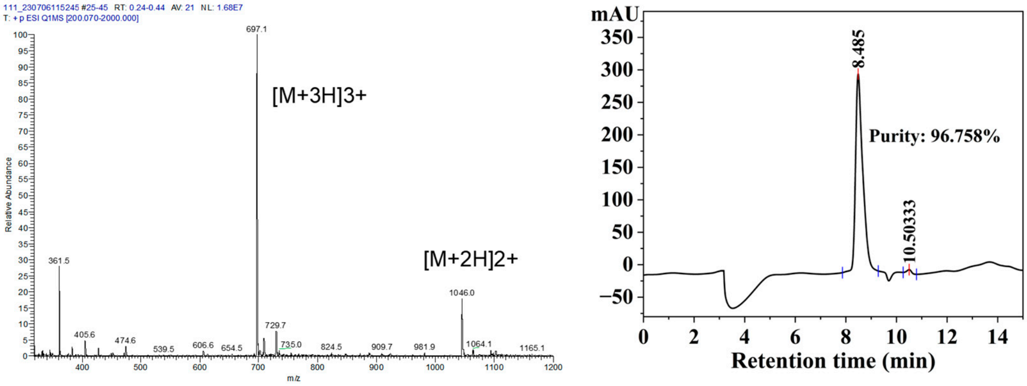

C2CL5

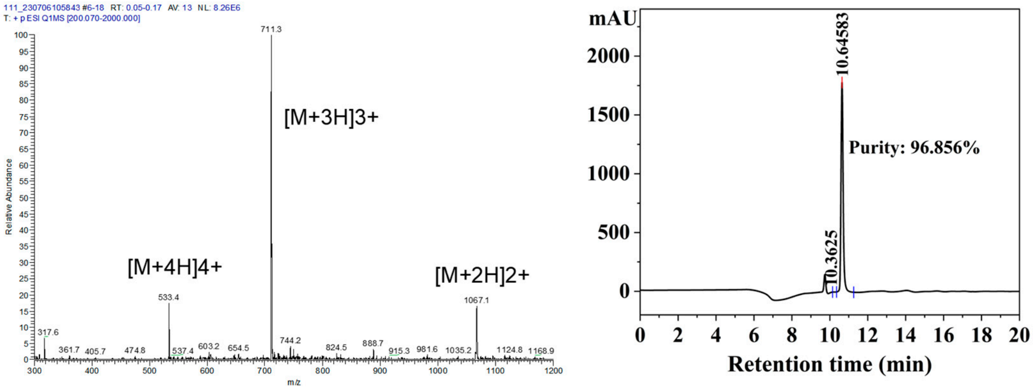

C4CL5

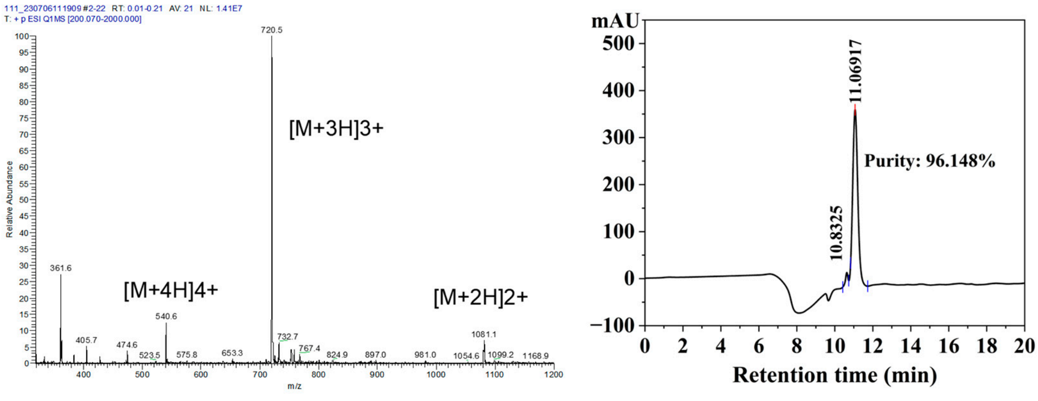

C6CL5

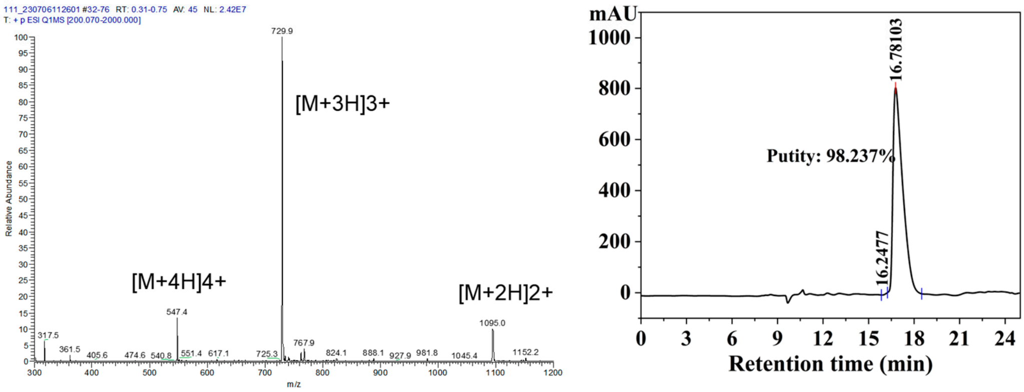

C8CL5

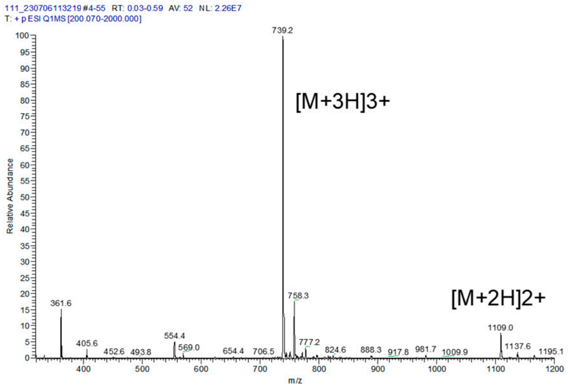

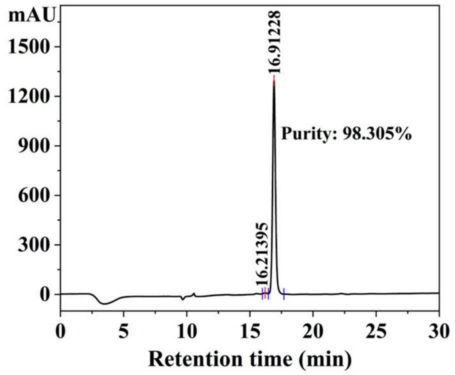

C10CL5

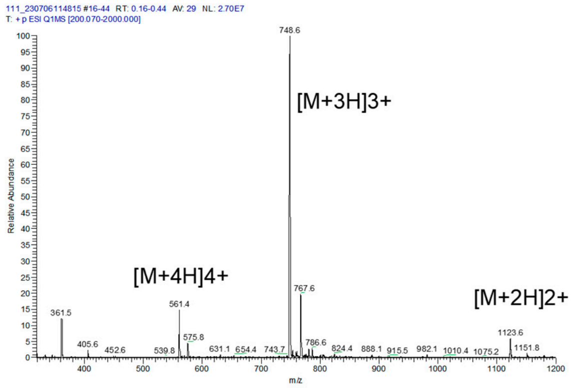

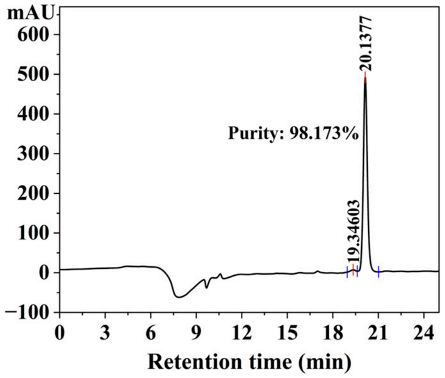

C12CL5

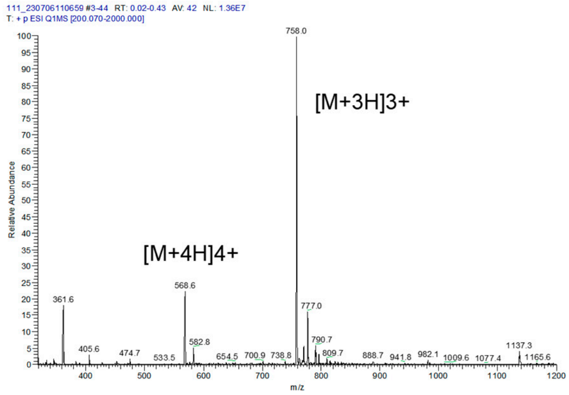

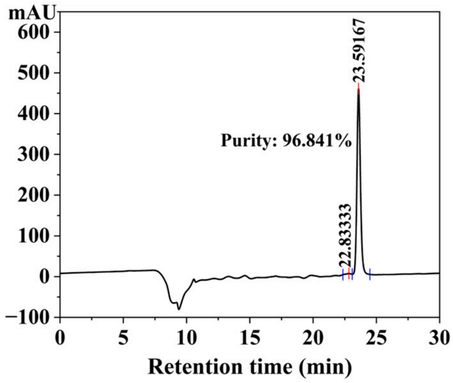

C14CL5

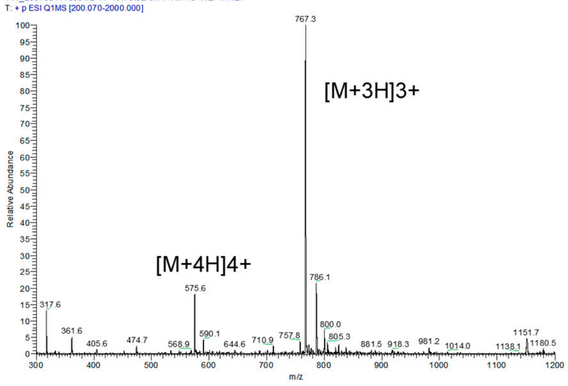

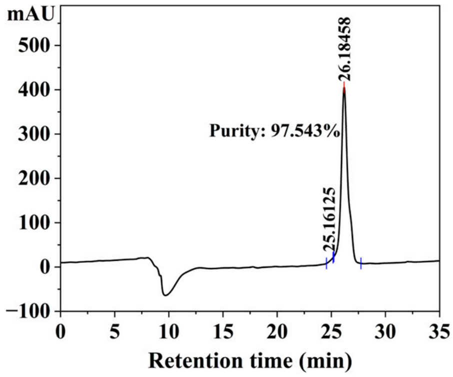

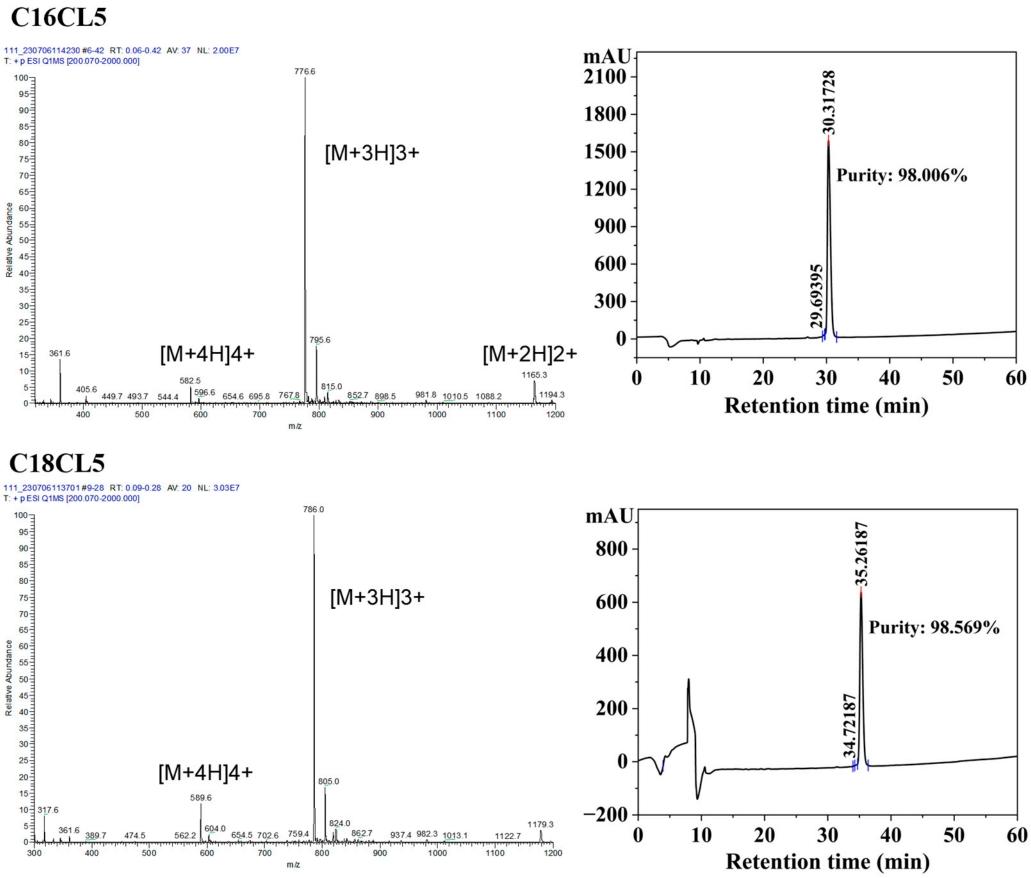

Figure S2. ESI-MS (left) and RP-HPLC (right) spectra of the lipopeptides.

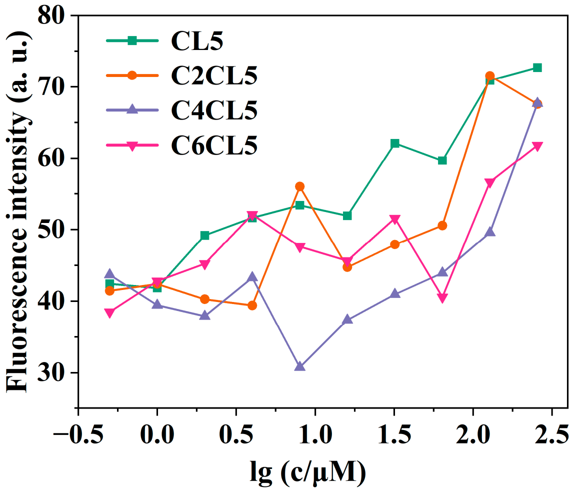

Figure S3. The fluorescence intensity of 1,8-ANS against the logarithmic values of lipopeptide concentrations.

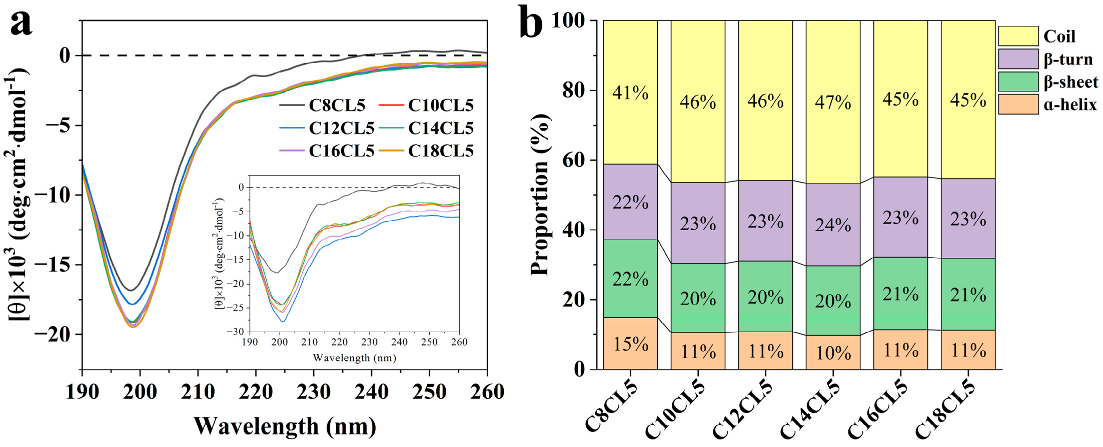

Figure S4. (a) CD spectra of C8CL5−C18CL5 at 100μM; the inset shows the CD spectra of C8CL5−C18CL5 at 6 μM. (b) Estimation of secondary structure proportions of C8CL5−C18CL5 at 100 μM using CDpro.

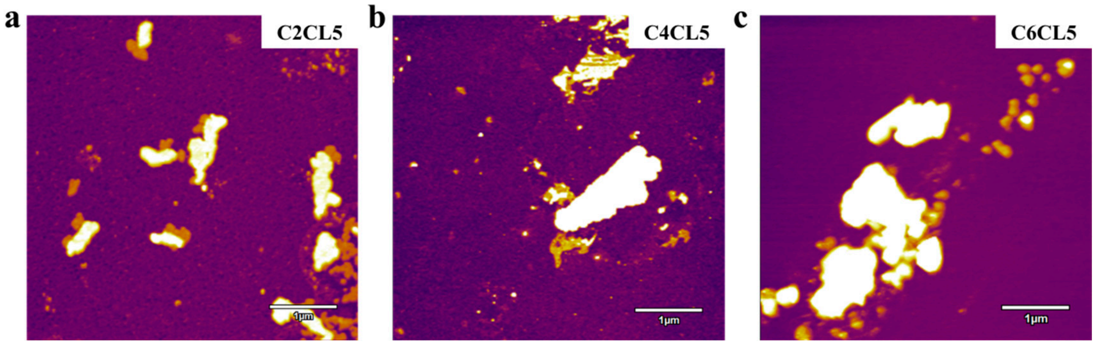

Figure S5. AFM images of (a) C2CL5, (b) C4CL5 and (c) C6CL5.

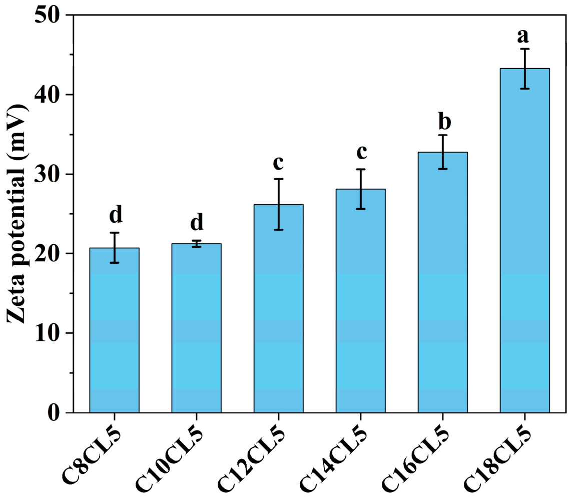

Figure S6. Zeta potentials of C8CL5−C18CL5. The measurements were repeated three times, and the data are expressed as the mean ± SD.

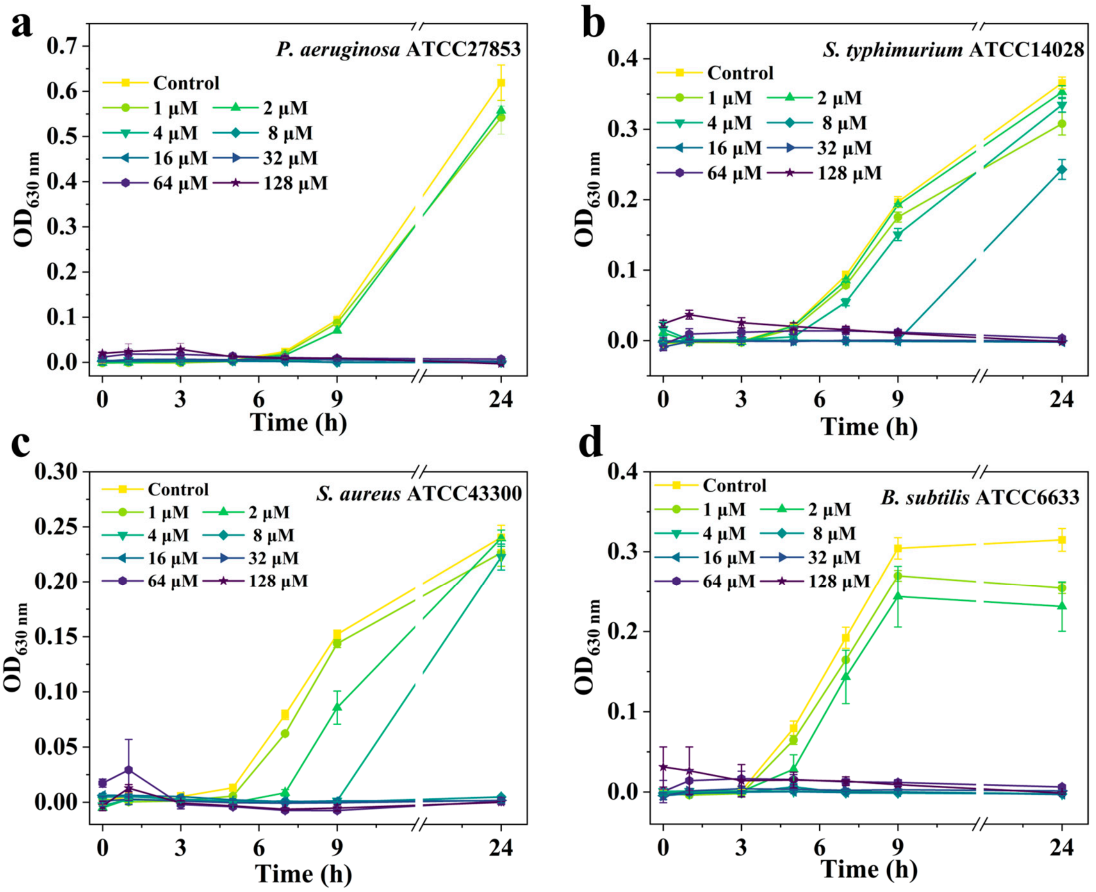

Figure S7. Effect of different concentrations of C10CL5 on the growth curves of *P. aeruginosa* ATCC27853 (a), *S. typhimurium* ATCC14028 (b), *S. aureus* ATCC43300 (c), *B. subtilis* ATCC6633 (d). The measurements were repeated three times, and the data are expressed as the mean ± SD.

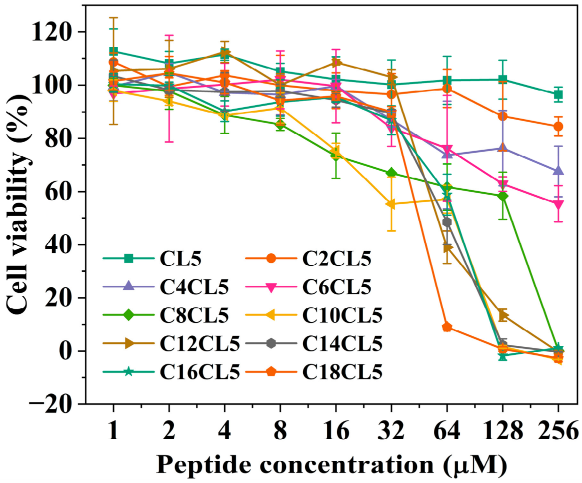

Fugure S8. Effects of lipopeptides at different concentrations on the viability of HEK-293 cells. The measurements were repeated three times, and the data are expressed as the mean ± SD.

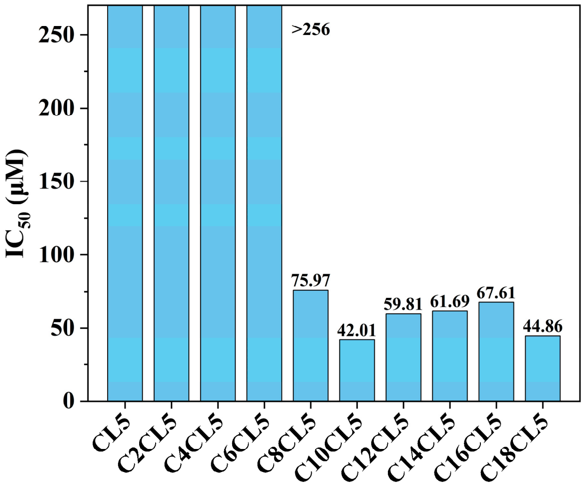

Fugure S9. The IC_{50} values of the lipopeptides toward HEK-293 cells.

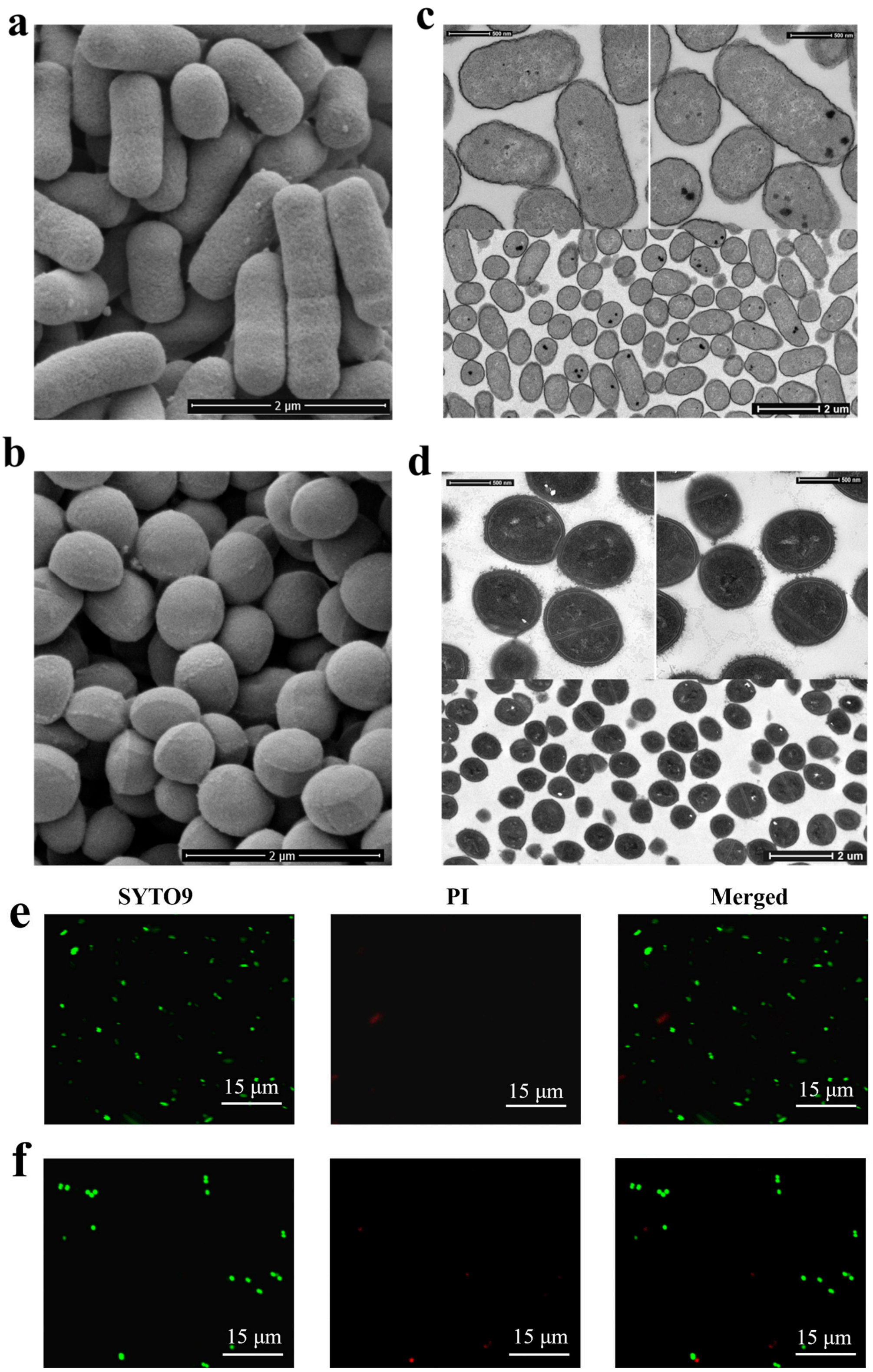

Figure S10. SEM (a, b), TEM (c, d) and CLSM (e, f) images of the untreated *E. coli* ATCC25922 (a, c, e) and *S. aureus* ATCC25923 (b, d, f).
